# Superintending an Operation

**Published:** 1900-07

**Authors:** 


**Superintending an Operation.**—A good one comes from Galveston. The doctors down there are "catching on,"—learning how to charge. A big operation was to be performed on a member of Mr. Richfellow's family. Dr. Sly, a distinguished and very able surgeon, was the "family physician." He diagnosed the case and advised the operation. Mr. Richfellow's wife was a second cousin, or something, to Dr. Guy's wife's aunt's stepson's wife, and she wanted Dr. Guy, also a very eminent and able surgeon, to do the operation. What she says goes, and Richfellow, he pays the freight,—no, the bills. To save Dr. Sly's feelings in the matter, and to "have him in it," Mr. Richfellow called and explained the situation to him, but added "we want you to be present and sorter superintend the operation." Operation a brilliant success. Dr. Guy calls on Dr. Sly, all smiles, and says: "Sly, I got $1500 for that op.; how much do you think I ought to shell out to you?" "Oh, nothing at all," says Sly, "I sent in my bill for $3000 for superintending the operation, and have just received a check for the money. It's a cold day," he said, wiping the perspiration from his brow, and with a faraway look, thoughtfully added, "when I get left."

